# Hysteretic Behavior of Random Particulate Composites by the Stochastic Finite Element Method

**DOI:** 10.3390/ma12182909

**Published:** 2019-09-09

**Authors:** Damian Sokołowski, Marcin Kamiński

**Affiliations:** Faculty of Civil Engineering, Architecture and Environmental Engineering, Łódź University of Technology, Al. Politechniki 6, 90-924 Łódź, Poland

**Keywords:** particulate composites, hyper-elasticity, hysteresis, stochastic perturbation technique, homogenization method

## Abstract

Hysteretic behavior of random particulate composite was analyzed using the stochastic finite element method and three independent probabilistic formulations, i.e., generalized iterative stochastic perturbation technique of the tenth order, Monte-Carlo simulation, and semi-analytical method. This study was based on computational homogenization of the representative volume element (RVE), and its main focus was to demonstrate an influence of random stress in constitutive relation to the matrix on the deformation energies stored in the effective (homogenized) medium. This was done numerically for an increasing uncertainty of random matrix admissible stress with a Gaussian probability density function, for which the relations to the energies of the entire composite were approximated via the weighted least squares method algorithm. This composite was made of two phases, a hyper-elastic matrix exhibiting hysteretic behavior and a linear elastic spherical reinforcing particle located centrally in the RVE. The RVE was subjected to a cyclic stretch with an increasing amplitude, and computations of deformation energies were carried out using the finite element method system ABAQUS. A stress–strain history of the homogenized medium has been presented for the extreme and for the mean mechanical properties of the matrix to illustrate the random hysteresis of the given composite. The first four probabilistic moments and coefficients of the RVE deformation energy were determined and have been presented in addition to the input statistical scattering of the admissible stresses.

## 1. Introduction

Modeling of multi-phase composites is a very demanding and complex challenge because of the multiple scales in composites, relatively high anisotropy, and many sources of uncertainty in the material and geometrical parameters. This is why a multiscale approach [1] has been widely applied in contemporary computational mechanics. One of the most common implementations involves the homogenization method, which employs the representative volume element (RVE), effectively describing the composite material at its macro scale. An exemplary problem of such an approach was applied in Reference [2], where three different geometrical scales included interfacial defects, interphase, and the entire RVE. The defects constituted a micro level and were further aggregated into an interphase around a particle, which when included in the RVE enabled computation of the effective stochastic stiffness tensor of this composite. Bear in mind that the first approaches to homogenization problems started from the linear elastic and isotropic response of the constituents, and exact periodicity of the RVE was required to obtain any analytical solution [3,4,5]. Elasticity of composites is still considered for several problems, as, for instance, a packing effect [6] or second strain gradient theory of elasticity [7], but it is now done with the aid of numerical approaches. The composites in nonlinear regimes are also numerically modeled [8], including multi-particle RVEs even with very complex micro-geometry, where analytical solutions are simply unavailable; accurate generation [9] and the size effect [10] in FEM models has recently become a research topic to minimize numerical error in the homogenization procedure. A leading method for homogenization in continuous materials is still the traditional finite element method (FEM), due to accessibility of academic or commercial software and the relatively small modeling error, where 2D and 3D first or second order finite elements play a crucial role. Other methods are also considered, such as some meshfree formulations [11], fast Fourier transform (FFT)-based methods [12], or even the discrete element method [13], which seems to be perfect for the cases where reinforcing or filling particles are densely packed into the matrix. There also exist plenty of various approaches for reducing the effort of FEM computations at the expense of accuracy. These aim to achieve a proper balance between the two mentioned factors for a specific multiscale problem. Some examples include clustering of the heterogeneous medium to several subdomains [14], usage of the artificial neural networks, machine learning approaches [15,16], and reduction of dimensionality of microscopic strain fields by manifold-learning methods [17]. A very contemporary problem under extensive research is the hyper-elastic response of elastomeric composite materials [18], especially for incompressible or almost incompressible composite solids with a huge contrast of mechanical properties between their components, where even an elastic reversible response is a complex problem [19]. Homogenization of composite materials with some nonlinearities is especially challenging in cases of some uncertainty source, and may be tackled with the use of stochastic [20,21] or polynomial chaos [22] approaches, where in addition to a complex mechanical behavior, a variety of random parameters are considered in the internal composition of the RVE or the constitutive law itself; some heat or electrical conductivity problems are still considered [23]. Therefore, the main objective of this study was to analyze a particle-reinforced composite with a hyper-elastic matrix (filler) and linearly elastic particle reinforcement, where both materials are isotropic. The first four probabilistic moments and coefficients of the deformation energy stored in the RVE of this composite under uniform cyclic stretch were computed. Numerical experiments were carried out here using the FEM system ABAQUS and computer algebra system MAPLE, where all probabilistic computations, together with the weighted least squares method fittings, were done. Probabilistic hysteresis and deformation energies were determined, starting from randomization of the matrix admissible stresses according to the Gaussian distribution, which showed that effective stiffness of this composite cannot be modeled by the Gaussian probability distribution function. 

## 2. Governing Equations

Let us consider a heterogeneous and continuous solid body Ω, and let as assume that this body is composed of two distinct components—a linear elastic reinforcement occupying the domain $\Omega_{p}$ and a hyper-elastic matrix contained in the region $\Omega_{m}$, where neither geometrical nor material imperfections are considered, so that

(1) $$\Omega =\Omega_{m}+\Omega_{p}$$

The representative volume element (RVE) includes a single spherical particle centrally located into the cubic volume of the matrix. A contact between the two components is perfect and ensured by a surface-based tie constraint, which eliminates the nodes on the slave surface with multi-point constraints. Interfacial defects may, however, be introduced with relatively small additional expense. This could be done by positioning the third thin phase in between the two main ones and varying its elastic properties, as in Reference [24]. A strain energy of the reinforcing particle phase can be computed as follows:

(2) $$U^{p}=\mu \varepsilon ÷\varepsilon +\frac{\lambda }{2}\;{\left(tr\left(\varepsilon \right)\right)}^{2}$$

where $\varepsilon =\frac{1}{2}\left(\nabla u+\nabla u^{T}+\nabla u\nabla u^{T}\right)$ denotes the strain tensor, and where the coefficients λ and μ are Lame constants and are related to the Young modulus Y and Poisson ratio ν:

(3) $$\mu^{L}=\frac{Y}{2\left(1+E\right)},\lambda^{L}=\frac{Y\nu }{\left(1+\nu \right)\left(1-2\nu \right)}$$

The first Piola–Kirchoff stress was then calculated here as $\bar{\sigma }=2\mu^{L}\varepsilon +\lambda^{L}tr\left(\varepsilon \right)I$. Next, the hyper-elastic strain energy in the matrix was defined by the van der Waals model:

(4) $$U^{m}=\mu^{L}\left\{-\left(\lambda_{m}^{L2}-3\right)\left[ln\left(1-\eta \right)+\eta \right]-\frac{2}{3}a\left(\frac{\tilde{I}-3}{2}\right)\right\}+\frac{1}{D}\left(\frac{J_{el}^{2}-1}{2}-ln\left(J_{el}\right)\right)$$

where $\tilde{I}=\left(1-\beta^{V}\right)\bar{I_{1}}+\beta^{V}\bar{I_{2}}$, $\eta =\sqrt{\frac{\tilde{I}-3}{\lambda_{m}^{L2}-3}}$, and where $\left[I_{1},\;I_{2}\right]$ are of course the first and the second deviatoric strain invariants. The entire deformation process has been considered here at constant room temperature, and the hysteretic behavior is governed by the following Equation:

(5) $${\dot{\epsilon }}_{B}^{cr}=A{\left[\lambda_{B}^{L,cr}-1+Y\right]}^{C}{\left(\sigma_{B}\right)}^{m}$$

where ${\dot{\epsilon }}_{B}^{cr}$ is the effective creep strain rate in network B, $\lambda_{B}^{L,cr}-1$ defines the nominal creep strain, and $\sigma_{B}$ represents the effective stress in this network. 

The effective constitutive tensor makes the deformation energies of the real and homogenized RVE equal to each other, so that

(6) $$\int_{\Omega }C_{\alpha \beta \chi \delta }^{}\varepsilon_{\alpha \beta }^{'}\varepsilon_{\chi \delta }^{'}d\Omega =\int_{\Omega }C_{\alpha \beta \chi \delta }^{\left(eff\right)}\varepsilon_{\alpha \beta }^{}\varepsilon_{\chi \delta }^{}d\Omega ,\;\;\;\;\alpha ,\beta ,\chi ,\delta =1,2,3$$

The strain energy of a real composite could be decomposed into that of the matrix and that of the particle, and, also, into the elastic (*el*) and creep-dissipation (*cd*) counterparts, and therefore:

(7) $$\int_{\Omega }C_{\alpha \beta \chi \delta }^{\left(eff\right)}\varepsilon_{\alpha \beta }^{'}\varepsilon_{\chi \delta }^{'}d\Omega =\int_{\Omega }C_{\alpha \beta \chi \delta }^{\left(p\right)}\varepsilon {'}_{\alpha \beta }^{}\varepsilon {'}_{\chi \delta }^{}d\Omega +\overset{}{\underset{\Omega }{\int }}C_{\alpha \beta \chi \delta }^{\left(m\right)}\varepsilon {'}_{\alpha \beta }^{}\varepsilon {'}_{\chi \delta }^{}d\Omega =U_{el}^{\left(p,m\right)}+U_{cd}^{\left(m\right)}$$

A statistical dispersion of maximum admissible stress in the matrix $\sigma_{11,max}$ was considered further, and such an approach was applied here to represent an uncertainty in matrix strength by a single equivalent parameter. This choice was well motivated by the experimental techniques, where one may exactly adjust the deformation and its rate, but the given material response may fluctuate in some uncertain way, which may be bounded by the hysteresis limits. It enabled relatively easy interpretation of this hysteretic behavior of the entire composite (Figure 4), which may periodically continue within the interval [$E\left(\sigma_{11,max}\right)-3\sigma \left(\sigma_{11,max}\right),E\left(\sigma_{11,max}\right)+3\sigma \left(\sigma_{11,max}\right)$]; σ stands here for the standard deviation and E for the expected value of the input uncertain parameter due to the assumed Gaussian probability distribution function of this admissible stress:

(8) $$p_{\sigma_{11,max}}\left(\sigma_{11,max}\right)=\frac{1}{\sigma \left(\sigma_{11,max}\right)\sqrt{2\pi }}exp\left(-\frac{{\left(\sigma_{11,max}-E\left(\sigma_{11,max}\right)\right)}^{2}}{2\sigma^{2}\left(\sigma_{11,max}\right)}\right);\sigma_{11,max}\in R$$

This particular choice of the input probability density function (PDF) came from the relatively wide usage of this function in various engineering applications. Furthermore, we recalled the following definitions of the probabilistic moments and coefficients as well as their statistical estimators (where *M* is the total number of random realizations and $p_{w}$ is the probability density function for the random parameter):

- expected value:
  (9) $$E\left(\sigma_{11,max}\right)=\int_{-\infty }^{+\infty }\sigma_{11,max}p_{w}\left(\sigma_{11,max}\right)dx\equiv \frac{1}{M}\overset{M}{\underset{i=1}{\sum }}\sigma_{11,max}{}^{\left(i\right)}$$
- variance of this stress:
  (10) $$Var\left(\sigma_{11,max}\right)=\int_{-\infty }^{+\infty }{\left(\sigma_{11,max}-E\left(\sigma_{11,max}\right)\right)}^{2}p_{w}\left(\sigma_{11,max}\right)dx\;\equiv \frac{1}{M-1}\overset{M}{\underset{i=1}{\sum }}{\left(\sigma_{11,max}-E\left[\sigma_{11,max}\right]\right)}^{2}$$
- coefficient of variation:
  (11) $$\alpha \left(\sigma_{11,max}\right)=\frac{\sqrt{Var\left(\sigma_{11,max}\right)}}{E\left(\sigma_{11,max}\right)}$$
- skewness β and kurtosis κ could be computed in the following way:
  (12) $$\beta \left(\sigma_{11,max}\right)=\frac{\mu_{3}\left(\sigma_{11,max}\right)}{\sigma^{3}\left(\sigma_{11,max}\right)};\;\kappa \left(\sigma_{max}\right)=\frac{\mu_{4}\left(\sigma_{11,max}\right)}{\sigma^{4}\left(\sigma_{11,max}\right)}-3$$
  where $\mu_{n}$ signifies the n^th^ central moment.

Obviously, an uncertainty of this stress induces in turn the statistical dispersion of the composite response and the computed energies of hyper-elastic stretch, which were the main objective of this study. A probabilistic framework of these computational experiments was completed using the iterative stochastic finite element method (ISFEM) according to the 10th order Taylor expansion [25], and was based upon the 6th order polynomial response function of the elastic and dissipated energies in relation to the input uncertain parameter $\sigma_{11,max}$. In this framework, the objective function was first formulated. It was aimed at connecting the uncertain parameter with a variable of which the stochastic characteristics are sought (here, the energies of the RVE). This connection could be either purely analytical, e.g., the constitutive relation, or it could be represented in a form of a response function. Since analytical connection between the sought variable and the uncertain parameter could be rarely established, within the ISFEM framework, a response function is preferred. This function was founded on the basis of numerical data, where the finite element method (FEM) is used in a way close to a parametric study. It is run with respect to the uncertain parameter in direct vicinity of its expected value, usually ±5% or ±10% from this value, with an equal spacing between each realization. The discrete results of the FEM are replaced with a continuous relation by means of a function of a specified class—a polynomial (called here the response function); other classes of function could also be used and the FEM could be replaced by a set of laboratory results or boundary element method results, to name a few. Further, this function was replaced with its Taylor expansion:

(13) $$U\left(\sigma_{11,max}\right)=U^{0}\left(\sigma_{11,max}{}^{0}\right)+\varepsilon \frac{\partial U\left(\sigma_{11,max}\right)}{\partial \sigma_{11,max}}{|}_{\sigma_{11,max}=\sigma_{11,max}{}^{0}}\Delta \sigma_{11,max}+\ldots \;+\frac{\varepsilon^{n}}{n!}\frac{\partial^{n}U\left(\sigma_{11,max}\right)}{\partial \sigma_{11,max}{}^{n}}{|}_{\sigma_{11,max}=\sigma_{11,max}{}^{0}}\Delta \sigma_{11,max}{}^{n}$$

where n stands for the order of expansion and also for an order of the ISFEM. Parameter ε is the perturbation coefficient, $U^{0}\left(\sigma_{11,max}{}^{0}\right)$ constitutes the expected value of the uncertain parameter, and the $n^{th}$ order variation is following $\varepsilon^{n}\Delta \sigma_{11,max}{}^{n}$.

The expected value of the uncertain parameter is calculated iteratively in the following manner:

(14) $$E\left(U\right)=U^{0}\left(\sigma_{11,max}{}^{0}\right)+\frac{\varepsilon^{2}}{n!}\frac{\partial^{2}U\left(\sigma_{11,max}\right)}{\partial \sigma_{11,max}{}^{2}}\mu_{2}\left(\sigma_{11,max}\right)+\ldots \;+\frac{\varepsilon^{n}}{n!}\frac{\partial^{n}U\left(\sigma_{11,max}\right)}{\partial \sigma_{11,max}{}^{2}}\mu_{n}\left(\sigma_{11,max}\right)$$

until a convergence of $U\left(\sigma_{11,max}\right)$.

Since the Taylor expansion converges with an increase of terms, accuracy of the ISFEM also increases with an increase of the order of this expansion. This happens at the expense of computational cost. The central moment for the Gaussian PDF has a closed form, different for even and odd orders of this moment p, which has a following form:

(15) $$\mu_{p}\left(\sigma_{11,max}\right)=\{\begin{array}{c} 0;p=2k+1\\ \left(\sigma {\left(\sigma_{11,max}\right)}^{p}\left(p-1\right)!!;p=2k\right) \end{array}$$

The closed form formulae of the higher order characteristics of the ISFEM for various PDFs, together with a detailed description of this approach, are available in References [25,26]. The major gain of the ISFEM in relation to the purely analytical approach consisting of direct integrations is the replacement of this apparatus with derivations, which are computationally inexpensive, and, what is even more important, always exist.

The expected values, coefficients of variation, skewness, and kurtosis computing using this technique were validated by Monte-Carlo simulation, and, independently, with the semi-analytical technique. These techniques are both based on the same polynomial as the ISFEM. The semi-analytical technique consists of a symbolic integration according to the aforementioned probability theory definitions. Its major limitation is the possibility of integration of this response, or objective function being one of the reasons for choosing a class of polynomials for the response function. The major shortcoming of the Monte-Carlo simulation is the computational cost required for an acceptable convergence—usually more than 100,000 trials.

## 3. Composite Material Model

**Figure 1:**
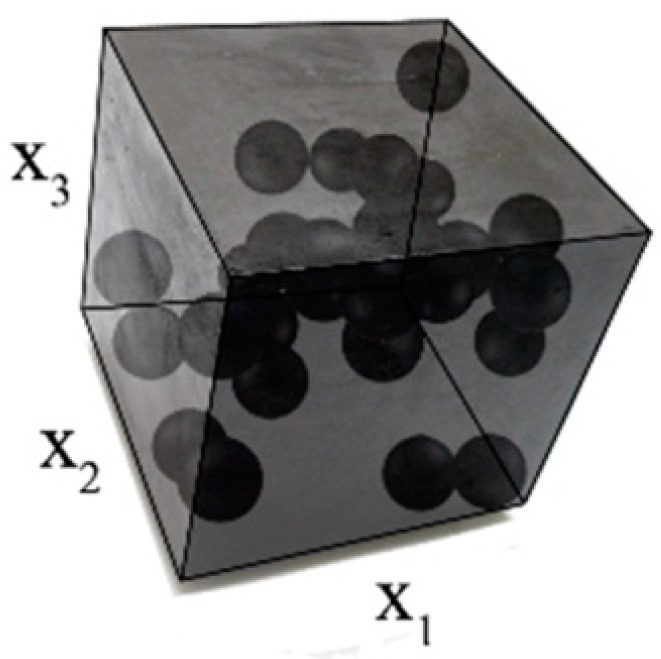
Internal structure of the composite.

**Figure 2:**
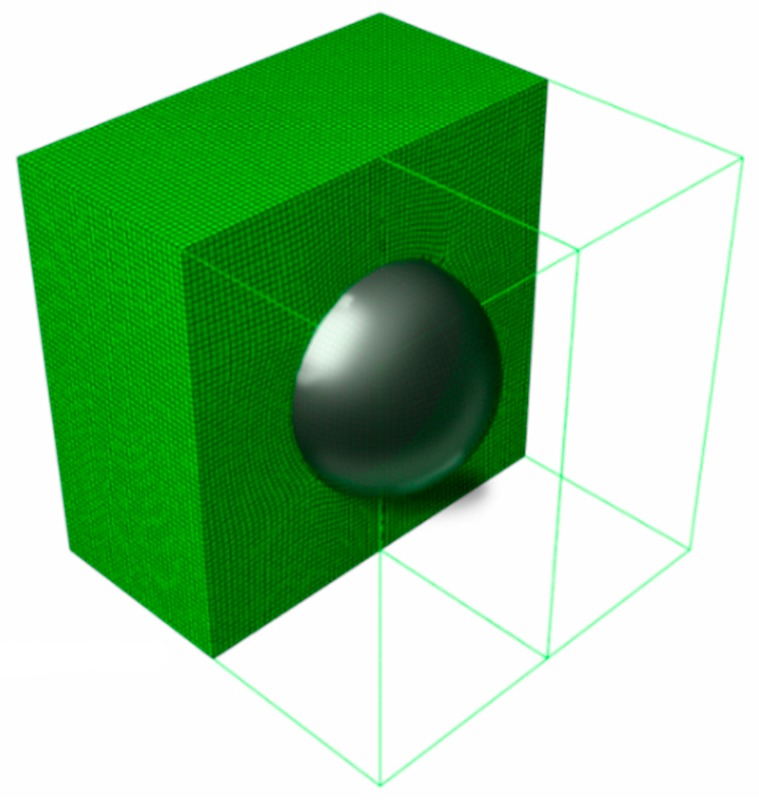
Representative volume element (RVE) of the composite.

**Figure 3:**
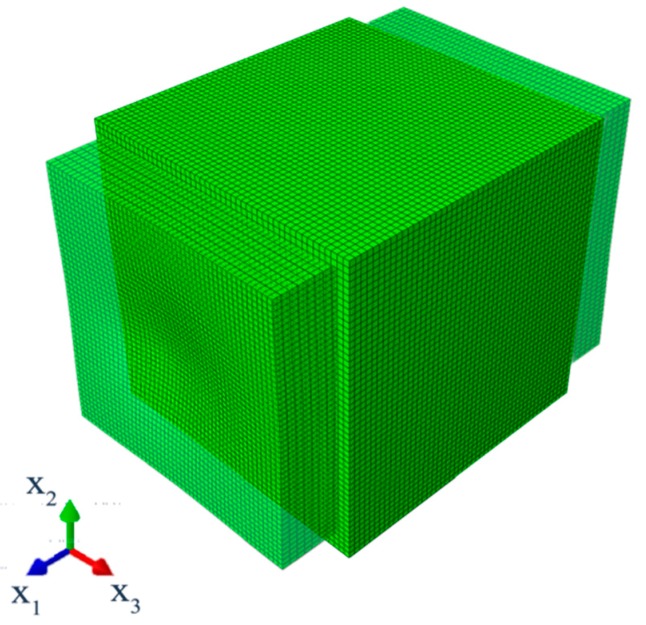
Discretization and deformation map of the composite RVE.

**Figure 4:**
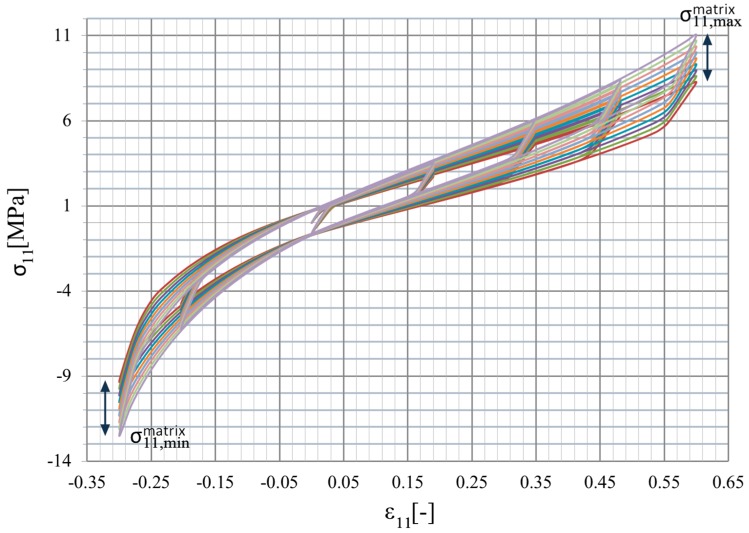
Hysteresis dispersion of the given composite matrix.

Determinations of the uncertainty level in the strain, dissipated, and internal energies of the particulate composite subjected to biaxial cyclic stretch were carried out for the hexagonal representative volume element (RVE) of the hyper-elastic two-phase composite made from Laripur LPR5020 filled with C60 fullerenes powder of 99.5% purity. The micro-structure of this composite is presented in Figure 1, where particles are randomly distributed within a spatial sample. A numerical model of this composite with a single spherical particle located centrally in the RVE is shown in Figure 2. This particle is linearly elastic and its material parameters are equal to Y=10GPa and μ=0.3, while the second component is the hyper-elastic polymeric matrix occupying 95% of the RVE. It has parameterized material properties $\chi_{i}=\left[\mu_{i},\lambda_{m,\;i},a_{i},\beta_{i},D_{i}\right],\;i\in 1,\;2\ldots ,\;11$, which correspond to a set of uncertain $\sigma_{11,max,i}$ with a mean value of 9.5 MPa. The hysteretic properties of the matrix include the stress scaling factor S=1, creep parameter A=0.1225, effective stress exponent m=1, and creep strain exponent c=−1. Numerical FEM model together with a principle deformation mode is given in Figure 3. Minimum, maximum and intermediate stress–strain curves for this matrix are presented in Figure 4, and they illustrate a spectrum of uncertain response of this matrix under the uniaxial stretch of the given RVE with free perpendicular surfaces, which is governed by the van der Waals hyper-elastic law.

**Figure 5:**
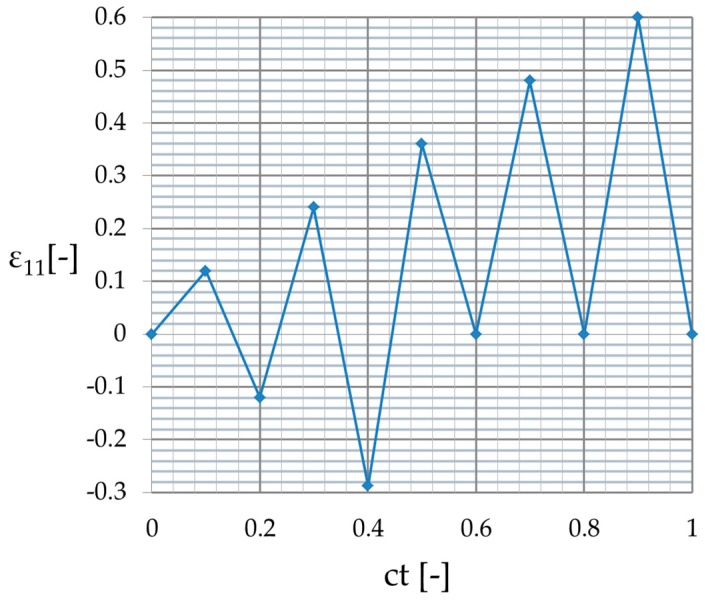
External strain applied on the outer edges of the RVE with respect to computation time ct.

The stretching level of the two-phase RVE applied in computations increased together within each of four stretch cycles until $\varepsilon_{11}=0.6$ and returned to zero at the end of this analysis. The detailed external strain cycles applied on the outer edges in biaxial tension are presented in Figure 5, and have been plotted versus the non-dimensional computational time ct, representing the entire deformation cycle in these computations. The boundary conditions applied included a fixed strain on two outer surfaces perpendicular to $x_{1}$ in direction $x_{1}$ with strain history according to Figure 5 and free perpendicular edges in direction $x_{2}$ and $x_{3}$. The finite element method computations were carried out in an implicit scheme in the ABAQUS 6.14 Standard system manufactured by Dassault Systèmes SE (more information on the algorithm could be found in Reference [27]). (Paris, France) The static general step procedure was used with approximately 50,000 20-noded quadratic brick elements, where C3D20 finite elements were applied to mesh the particle and the hybrid linear pressure C3D20H finite elements were used to discretize the matrix phase. This discretization was optimized during the initial numerical error verification to minimize the computational effort necessary for satisfactory efficiency of the resulting deformation energies. A direct sparse solver with multi-front technique was used and implemented together with the full Newton algorithm. The finite element method (FEM) equations are presented in the Appendix A for interested readers. The initial increment size was 0.0005, minimum allowed increment size was $10^{-5}$, and maximum increment size was 0.01. The field and history outputs were written in 860 equally spaced intervals along the time, 170 per cycle; time incrementation was set as automatic, where initial increment was applied and then its initial size was either reduced or increased based on the number of required equilibrium iterations for the current increment. Specifically, when fewer than 4 equilibrium iterations were required in two consecutive increments, the time increment was increased by a factor of 1.5, and when more than 10 iterations were required for the current increment, the next increment was reduced by factor of 0.75. The increment surpassing time of this interval was reduced to exactly the size required to match its time when numerical analysis got close to one of the space intervals where the outputs were expected.

**Figure 6:**
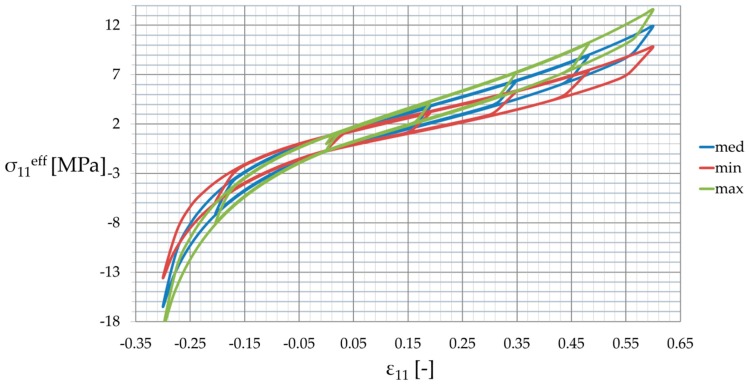
Effective response for the elastomer.

The computational experiment started from determination of the input parameters of the matrix’s hyper-elastic potential $\chi_{i}=\left[\mu_{i},\lambda_{m,\;i},a_{i},\beta_{i},D_{i}\right]$ by ensuring its response crossing through a set of 11 points $\sigma_{11,max,i}\left(\varepsilon_{11}=0.6\right),\;i\in \langle 1,\;2\ldots ,\;11\rangle$ corresponding to an uncertain parameters of this analysis. This was done by minimization of the following functions $\Phi_{i}\left(\mu_{i},\lambda_{m,\;i},a_{i},\beta_{i},D_{i}\right)=\sigma_{11,max,i}\left(\varepsilon_{11}=0.6\right)-\sigma_{11,i}\left(\varepsilon_{11}=0.6\right)$, where $\sigma_{11,\;i}\left(\varepsilon_{11}\right)$ was determined on a single FEM element, stress–strain history of the matrix presented in Figure 4. These material parameters remained constant for each discrete value $\sigma_{11,max,i}$. Furthermore, we proceeded with numerical determination of the energies in the homogenized medium—it was completed for the set of 11 discrete points $U\left(\sigma_{11,max}\right)$ about a mean value of $\sigma_{11,max}=9.5\;\mathrm{MPa}$, and was done for three various energies $U_{el}$, $U_{cd}$ and $U_{i}$. A set of $U\left(\sigma_{11,max}\right)$ was obtained together with the stress-strain histories of the RVE for all $\sigma_{11,i}^{eff}\left(\varepsilon \right)$ (see Figure 6), where the effective stress $\sigma_{11}^{eff}$ represents the average value calculated on its entire domain. Next, a discrete set of the energies was replaced with the continuous polynomial function by use of the weighted least squares method. These polynomials, included into the Taylor expansion of the 10^th^ order, served for a final calculation of the basic probabilistic characteristics of the energies in relation to the uncertain stress in the matrix $\left(\sigma_{11,max}\right)$, so that expectations, coefficients of variation, skewness, and kurtosis E(U), α(U), β(U), κ(U) were determined. Each characteristic was computed with use of three independent methods—stochastic perturbation, semi-analytical method, and Monte-Carlo simulation, and this was done for three various energies under consideration—elastic strain energy $U_{el}$, dissipation energy $U_{cd}$, and the total internal energy $U_{i}$. 

## 4. Numerical Results

**Figure 7:**
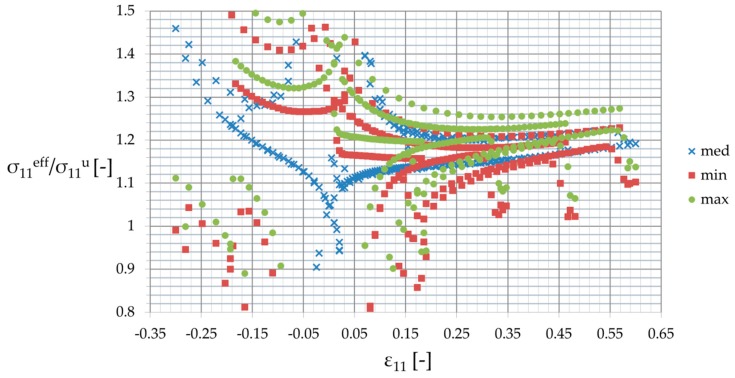
Strengthening ratios for the polymer after reinforcing.

First, the response of the homogenized reinforced polymer to the uniaxial external strain $\sigma_{11}^{eff}\left(\varepsilon_{11}\right)$ was considered and deterministic results have been discussed. The RVE was subjected to the same strain history as the pure matrix, and the results are reported in Figure 6 and Figure 7 for minimum (min), maximum (max), and mean (med) properties of the matrix resulting from a dispersion of $\sigma_{11,max}$. An increase of the matrix properties improved strengthening effectiveness of reinforcement η calculated as $\sigma_{11,max}^{eff}/\sigma_{11,max}$, from $\eta_{min}=1.2$ for the lowest $\sigma_{11,max}$ through to $\eta_{max}=1.28$ for the highest one (see Figure 6). Figure 7 shows the strengthening ratio of the composite calculated as a ratio of the reinforced polymer stress vs. the one for unreinforced polymer $\sigma_{11}^{eff}/\sigma_{11}^{u}$. This was calculated at a certain strain for minimum, maximum, and mean properties of the matrix for the entire strain history. It shows that this ratio was a little bit unpredictable for strains close to and less than 0, but in each case, it converged to a certain value towards the maximum strain and increased toward a 0 value. This means a steeper curve (higher stiffness) for the small strain region—$\varepsilon_{11}\leq 0.15$—than for the high strain region. Secondly, this ratio increased a little together with an increase of properties, and it depended on the strain history and the direction of loading (loading or unloading). 

**Figure 8:**
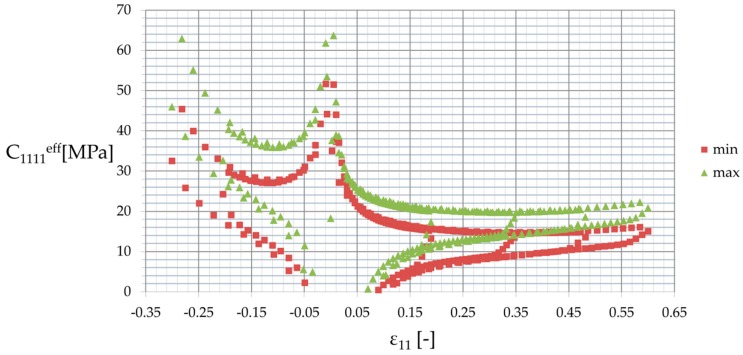
Effective stiffness of the polymer $C_{1111}^{eff}$ with respect to strain $\varepsilon_{11}$.

Furthermore, the composite stiffness defined as $\partial \sigma_{11}/\partial \varepsilon_{11}$ and denoted by $C_{1111}^{eff}$ is presented in Figure 8 for the smallest (min) and the largest (max) properties of the polymeric matrix. It shows a good convergence for the given loading cycles, except for the strains close to 0—$\varepsilon_{11}\in \left(-0.03\;,\;0.03\right)$,—where it increased or decreased to ±∞. This was caused by stress and strain variations being very close to 0, and, more importantly, by the computation accuracy. Figure 8 additionally shows a stabilization of this stiffness for relatively high strain levels, i.e., $\varepsilon_{11}>0.15$, where two clearly distinctive relations of both loading and unloading existed, reflecting hysteretic behavior. These relations were highly nonlinear around zero strain and linearized together with its increase—they became almost constant with respect to ε for positive strain, and increased together with its decrease. A difference of the loading to unloading curves decreased when the strain became more distant from 0, and, as expected, they preserved common points when loading ended and unloading began. Finally, it is of note that the effective stiffness tensor component $C_{1111}^{eff}$ was of course higher for the maximum material properties of the matrix than for its minimum values, and this difference reached 40% in some cases.

The final results dealt with the probabilistic part of the study, and included the first four probabilistic characteristics of the maximum values of three different energies, i.e., elastic strain energy $U_{el}$, dissipation energy $U_{cd}$, and the total internal energy $U_{i}$, calculated for the entire RVE and presented with respect to the input statistical scattering $\;\alpha \left(\sigma_{11,max}\right)$. They were calculated using three various and concurrent probabilistic techniques, i.e., iterative stochastic perturbation-based finite element method (SPT), crude Monte-Carlo simulation with 400,000 trials (MCS), and semi-analytical method (AM) implemented in the symbolic algebra system MAPLE 2017. As mentioned above, SPT and AM are based on the same polynomial response functions determined by the weighted least squares method (WLSM). These response functions, relating the deformation energy U with the Gaussian parameter $\sigma_{max}$, were obtained from a set of 11 equally spaced discrete FEM realizations of the RVE strain history in the space of $\sigma_{11,max}$. The discrete $\sigma_{11,max}$ values were within ±20% of the mean value of 9.5 MPa. The weights in the WLSM are of the Dirac type with magnitudes of w∈[1,1,1,1,1,11,1,1,1,1,1], so that the mean value has the highest impact on the polynomial basis coefficients. 

**Figure 9:**
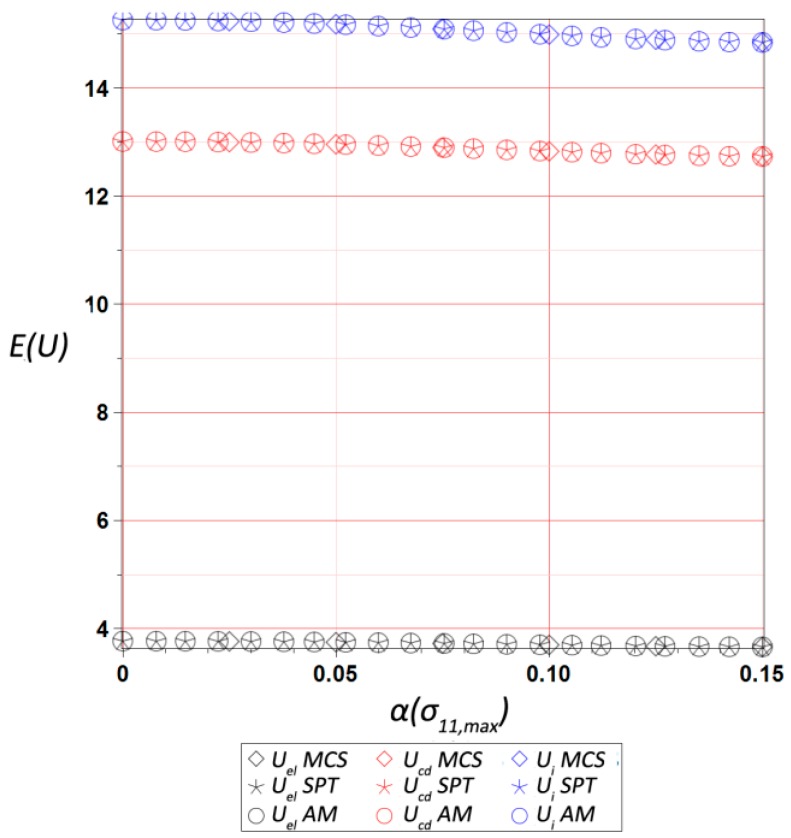
Expected value of the ultimate elastic strain $U_{el}$, dissipation $U_{cd}$, and internal $U_{i}$ energies in the RVE vs. $\alpha \left(\sigma_{11,max}\right)$.

**Figure 10:**
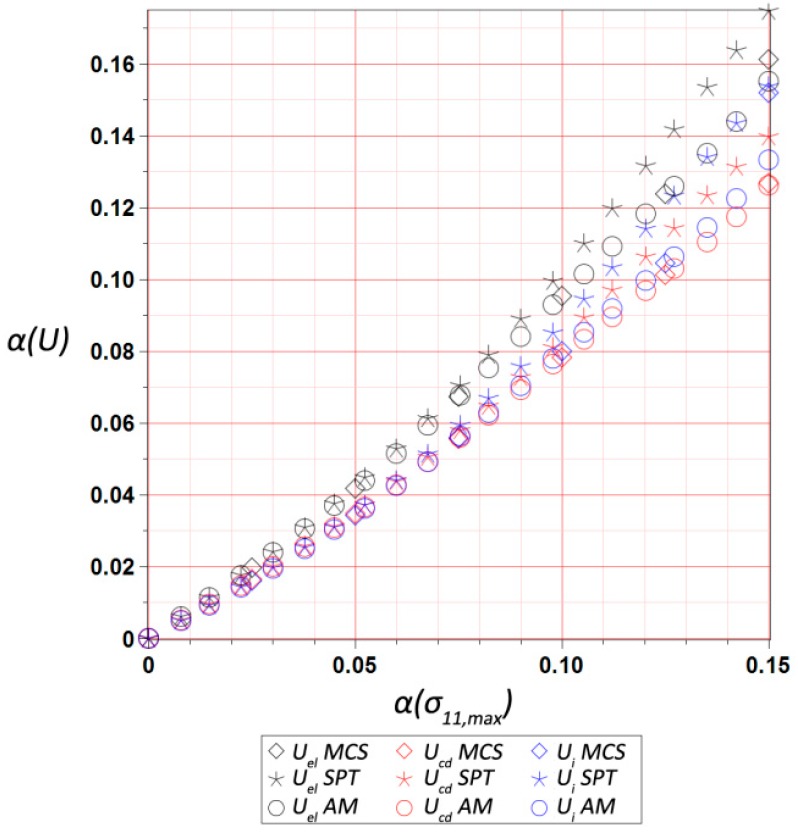
Coefficient of variation of ultimate elastic strain $U_{el}$, dissipation $U_{cd}$, and internal $U_{i}$ energies in the RVE vs. $\alpha \left(\sigma_{11,max}\right)$.

The expected values are presented in Figure 9, and show a moderate dependence on the $\alpha \left(\sigma_{11,max}\right)$, with a small decrease of all energies together with its increase. The internal energy expectation was the largest one, followed by the dissipated energy, while the elastic one was more than four times smaller. This is because of a cyclic stretch, where dissipated energy increased during each relaxation, while the elastic energy depended on the stretch level only. The internal energy as a sum of these two was the largest during the last cycle for the ultimate strain of 0.8, where elastic strain energy was maximized. During comparison of the output coefficients of variation collected in Figure 10, it was clearly seen that they were almost proportional and very close to the input uncertainty. The one for $U_{el}$ was the largest, while variation of $U_{cd}$ exhibited minimum value; quite obviously, this difference increased together with an increase of $\alpha \left(\sigma_{11,max}\right)$, and reached approximately 30% for $\alpha \left(\sigma_{11,max}\right)=0.15$. An output of the three concurrent probabilistic methods showed a perfect agreement for the expected values was is a little worse for the coefficients of variation; nevertheless, the results shown in Figure 10 were still very close to each other and within 5% tolerance. 

**Figure 11:**
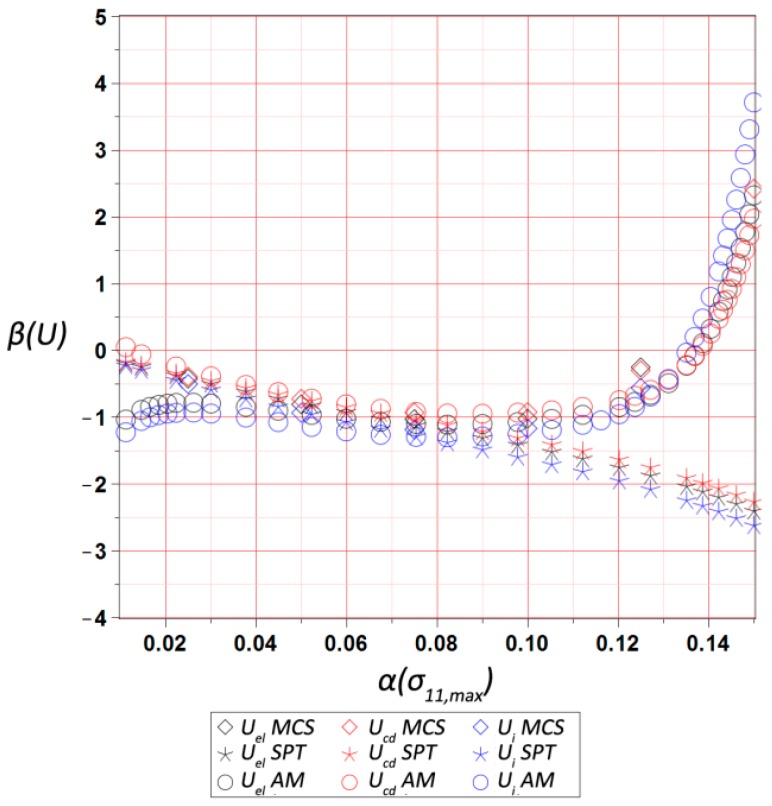
Skewness of the ultimate elastic strain $U_{el}$, dissipation $U_{cd}$, and internal $U_{i}$ energies in the RVE vs. $\alpha \left(\sigma_{11,max}\right)$.

**Figure 12:**
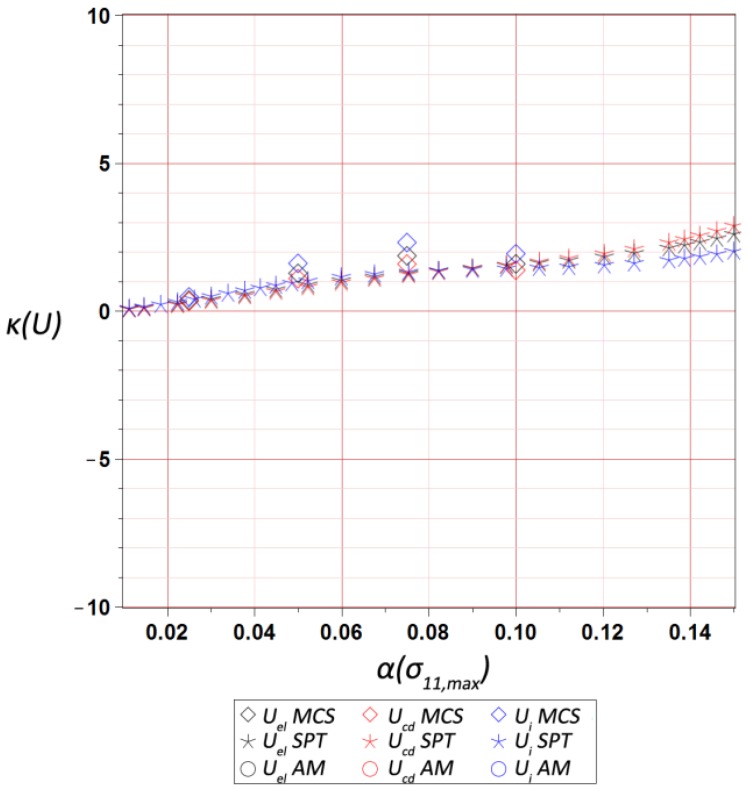
Kurtosis of ultimate elastic strain $U_{el}$, dissipation $U_{cd}$, and internal $U_{i}$ energies in the RVE vs. $\alpha \left(\sigma_{11,max}\right)$.

Skewness trends β(U) presented in Figure 11 were the most sensitive to the input uncertainty level—not only its magnitude, but the sign also depended upon the values of $\alpha \left(\sigma_{11,max}\right)$. This highly non-linear behavior resulted in a difficulty in precise determination of this parameter by any of the probabilistic methods, which accidentally returned similar value. It was additionally seen that the semi-analytical method returned relatively higher errors for smaller $\alpha \left(\sigma_{11,max}\right)$, while the SPT seemed to be inefficient for $\alpha \left(\sigma_{11,max}\right)>0.09$. This was because of a high complexity of analytical calculus in the AM technique, which may also have been a reason for its unavailability for determination of the kurtosis κ(U). This kurtosis κ(U) (collected in Figure 12) always moderately increased together with an additional increase of an input uncertainty level, and had a very similar magnitude for the SPT and MCS. Interestingly, its variations were much more linear than for the skewness, and at the same time very similar for all the energies. These two probabilistic characteristics remarkably differed from 0, so that the resulting probability distributions of internal energies under consideration were rather distant from the Gaussian bell-shaped distribution. Therefore, they could not be simply and directly approximated by their first two moments, and demanded significant computer power and higher order stochastic analyses using the techniques employed above.

## 5. Conclusions

Deformation energies in the hyper-elastic RVE of a composite subjected to a cyclic stretch demonstrated almost the same level of uncertainty as the input admissible stress in the hyper-elastic matrix $\sigma_{11,max}$. The analyzed expectations of deformation energies decreased a little bit together with an increase of an input uncertainty; all three considered energies of the homogenized medium were similarly affected by $\alpha \left(\sigma_{11,max}\right)$, whereas the skewness as well as kurtosis differed both from zero, demonstrating remarkable magnitudes and exhibiting that the resulting energy PDF was definitely non-Gaussian. As the linear combination of this energy was included in the effective material tensor, this tensor also exhibited non-Gaussian distribution in this case. This means that simple second order second moment (SOSM) probabilistic analysis is inefficient for such study, and generalized higher order stochastic study must be carried out to efficiently estimate the probabilistic moments of the homogenized material. This result for the hyper-elastic composite was totally different than the elastic one, where the resulting effective elasticity tensor was almost always Gaussian.

Moreover, it is necessary to underline that the correlation of the results coming from three independent probabilistic methods was almost perfect for E(U), very high for α(U), conditional for κ(U), and not fully achieved for β(U), where the semi-analytical method was ineffective for small input uncertainties and the stochastic perturbation method, i.e., for high statistical scattering. Further, we concluded that strengthening efficiency increased a little bit together with an increase of hyper-elastic matrix properties. Effective stiffness of the composite also increased with an increasing matrix properties, and it preserved two distinctive curves for loading and unloading. The relationship of this effective stiffness to the strain level was highly nonlinear about zero strain, and linearized together with its increase or decrease. The relationship of the maximum stress in pure polymer and in the reinforced polymer was nonlinear. Further research will concern the effect of $\alpha \left(\sigma_{11,max}\right)$ on the effective constitutive law of the homogenized medium, and also the effect of random interface defects on the hyper-elastic response of the effective medium in three-phase particulate composites consisting of matrix, reinforcement, and an interphase.

## Appendix A

Let us consider the following boundary-value problem, where the displacement function u is to be determined [28,29,30,31]:

(A1) $$\{\begin{array}{c} -div\left(\bar{\sigma }\left(u\right)\right)=f\;in\;\Omega \\ \begin{array}{c} u=\bar{u}\;on\;\Gamma_{D}\\ \bar{\sigma }\left(u\right)n=h\;on\;\Gamma_{N} \end{array} \end{array}$$

where suitable Dirichlet and Neumann boundary displacements $\bar{u}$ on $\Gamma_{D}$ and tractions h on $\Gamma_{N}$ are prescribed. A weak formulation necessary in the FEM is proposed as:

(A2) $$\int_{\Omega }\bar{\sigma }\left(u\right)\nabla wd\Omega =\int_{\Omega }f\;ud\Omega +\int_{\Gamma_{N}}h\;ud\Gamma$$

where ***w*** and ***u*** also belong to the additional kinematically admissible spaces. Further, the FEM complete partition of the domain Ω is provided, so that by introducing the finite element base functions $\varphi_{i}$ (with *N* denoting the total number of degrees of freedom in the model), one defines a vector the of internal forces:

(A3) $$f_{i}^{\mathrm{int}}\left(u\right)=\int_{\Omega }\bar{\sigma }\left(u\right)\;\nabla \varphi_{i}d\Omega ,\;i=1\ldots ,N$$

and also the vector of external forces:

(A4) $$f_{i}^{ext}\left(u\right)=\int_{\Omega }f\cdot \varphi_{i}d\Omega +\int_{\Gamma_{R}}h\cdot \varphi_{i}d\Gamma ,\;i=1\ldots ,N$$

So that Equation (9) can be represented as:

(A5) $$R\left(u\right)=f^{int}\left(u\right)-f^{ext}=0$$

and this nonlinear system of equations is solved with the use of the Newton method, where such δu is sought that the following Equation converges:

(A6) $$J\left(u^{k}\right)\delta u=-R\left(u^{k}\right)$$

J traditionally denotes the Jacobian matrix:

(A7) $$J\left(w\right)\delta u=\frac{\delta f^{int}}{\delta u}\left(u\right)$$

This was completed for consecutive steps k=1,2…, s until the desired convergence condition in the FEM experiments was met, where the additional criterion was provided a priori.
